# Inferring miRNA sponge co-regulation of protein-protein interactions in human breast cancer

**DOI:** 10.1186/s12859-017-1672-2

**Published:** 2017-05-08

**Authors:** Junpeng Zhang, Thuc Duy Le, Lin Liu, Jiuyong Li

**Affiliations:** 1 School of Engineering, Dali University, Dali, Yunnan 671003 People’s Republic of China; 2 School of Information Technology and Mathematical Sciences, University of South Australia, Mawson Lakes, SA 5095 Australia; 3 Centre for Cancer Biology, University of South Australia, Adelaide, SA 5000 Australia

**Keywords:** miRNA sponge, ceRNA, miRNA sponge co-regulation, lncRNA, Protein-protein interaction, Breast cancer

## Abstract

**Background:**

Recent studies have shown that the crosstalk between microRNA (miRNA) sponges plays an important role in human cancers. However, the co-regulation roles of miRNA sponges in protein-protein interactions (PPIs) are still unknown.

**Results:**

In this study, we propose a multi-step method called miRSCoPPI to infer miRNA sponge co-regulation of PPIs. We focus on investigating breast cancer (BRCA) related miRNA sponge co-regulation, by integrating heterogeneous data, including miRNA, long non-coding RNA (lncRNA) and messenger RNA (mRNA) expression data, experimentally validated miRNA-target interactions, PPIs and lncRNA-target interactions, and the list of breast cancer genes. We find that the inferred BRCA-related miRSCoPPI network is highly connected and scale free. The top 10% hub genes in the BRCA-related miRSCoPPI network have potential biological implications in breast cancer. By utilizing a graph clustering method, we discover 17 BRCA-related miRSCoPPI modules. Through pathway enrichment analysis of the modules, we find that several modules are significantly enriched in pathways associated with breast cancer. Moreover, 10 modules have good performance in classifying breast tumor and normal samples, and can act as module signatures for prognostication. By using putative computationally predicted miRNA-target interactions, we have consistent results with those obtained using experimentally validated miRNA-target interactions, indicating that miRSCoPPI is robust in inferring miRNA sponge co-regulation of PPIs in human breast cancer.

**Conclusions:**

Taken together, the results demonstrate that miRSCoPPI is a promising tool for inferring BRCA-related miRNA sponge co-regulation of PPIs and it can help with the understanding of the co-regulation roles of miRNA sponges on the PPIs.

**Electronic supplementary material:**

The online version of this article (doi:10.1186/s12859-017-1672-2) contains supplementary material, which is available to authorized users.

## Background

microRNAs (miRNAs) are a kind of single-stranded small non-coding RNA molecules (~22 nt) found in different organisms. They widely participate in many biological functions [1, 2]. Computational analysis estimates that >60% of human protein-coding genes are regulated by miRNAs through conserved base-pairing between the 3′-untranslated region (UTR), 5′ UTR, and open reading frames (ORF) of mRNAs and 5′ seed region of miRNAs [3]. In general, miRNAs cause inhibition of translation and/or degradation of mRNAs. Occasionally, miRNAs also positively regulate gene expression and/or increase translation of mRNAs [4, 5].

Recently, the hypothesis of competing endogenous RNA (ceRNA) has been proposed [6]. Based on the hypothesis, a pool of RNAs acting as ceRNAs (also called miRNA sponges), such as lncRNAs, pseudogenes, mRNAs and circular RNAs (circRNAs), compete with each other by sponging miRNAs for interactions. Previous studies have shown that miRNA sponges play important roles in the physiology and development of human cancers [7, 8].

Existing computational methods of investigating miRNA sponges can be divided into three categories: (1) miRNA sponge recognition, (2) identification of miRNA sponge interaction networks, and (3) miRNA sponge module exploration. In the first category [9, 10], similar to miRNA target recognition, the principle of complementary base-pairing is assumed when recognizing miRNA sponges. In other words, it is assumed that there is an interaction between the 5′-end of an miRNA called the ‘seed region’ and the sequence of the miRNA sponge. However, improperly designed miRNA sponge sequences may cause high false-positive miRNA sponge recognition. By integrating expression profiles and putative miRNA-target interactions, several *in silico* or mathematical models in the second category [11–16] have been proposed to identify miRNA sponge interaction networks. The identification of miRNA sponge interaction networks provides a global way to study the biological functions and regulatory mechanisms of miRNA sponges. Since modularity is an important property in cancer progression and development, the third category [17–19] focuses on exploring miRNA sponge modules to study module-level properties of miRNA sponges in cancer. The identified functional miRNA sponge modules could be regarded as potential module biomarkers in specific cancer, e.g., breast cancer and lung cancer.

The above work from different perspectives investigates the crosstalk between miRNA sponges. However, they don’t consider the co-regulation roles of miRNA sponges in protein-protein interactions (PPIs), which, actually, can help to understand how miRNA sponges influence the downstream biological processes. Proteins are the major functional units in living cells, and they rarely work alone. PPIs make up the protein interactome of organism, and are the basis of most biological processes. Moreover, understanding PPI networks can provide insight into the behaviour of cancer cells [20]. Consequently, inferring miRNA sponge co-regulation of PPIs could facilitate the understanding of biological mechanisms within living cells.

In this study, we propose a multi-step method to infer miRNA Sponge Co-regulation of PPIs (thus the proposed method is called miRSCoPPI). The method is applied to the breast invasive carcinoma (BRCA) dataset provided by The Cancer Genome Atlas (TCGA) to infer BRCA-related miRSCoPPI network. We firstly integrate matched miRNA, lncRNA and mRNA expression data, experimentally validated miRNA-target interactions, and the list of breast cancer genes to identify BRCA-related miRNA sponge interaction network. Next, we search for two types of pre-defined miRSCoPPI motifs in the network consisting of BRCA-related miRNA sponge interactions, PPIs and lncRNA-target interactions. By merging the identified miRSCoPPI motifs, we obtain the BRCA-related miRSCoPPI network. Further investigation into the topological properties of the BRCA-related miRSCoPPI network, we discover that the network is highly connected and scale free. Through cluster analysis of the BRCA-related miRSCoPPI network, we find 17 BRCA-related miRSCoPPI modules. Pathway enrichment analysis results show that several modules are enriched in pathways related to breast cancer. In addition, 58.82% (10 out of 17) of modules have good performance in classifying breast tumor and normal samples, and are regarded as module signatures for prognostication. Finally, miRSCoPPI is robust in inferring BRCA-related miRNA sponge co-regulation of PPIs.

## Methods

### Data sources

We obtain the matched miRNA, lncRNA and mRNA expression profiles of human breast cancer (BRCA) from Paci et al. [21]. We use the *biomaRt* [22] Bioconductor package for gene ID conversion. The lncRNAs and mRNAs without gene symbols are removed, and the unique expression values of replicate miRNAs and mRNAs are obtained by taking the average expression value. As a result, we have expression profiles of 453 miRNAs, 470 lncRNAs and 11157 mRNAs. Samples of BRCA categorized as tumor (72 samples) and normal (72 samples) are used in this work.

The experimentally validated miRNA-target interactions consist of two types: miRNA-mRNA interactions and miRNA-lncRNA interactions. The miRNA-mRNA interactions are obtained by integrating miRTarBase v6.1 [23], TarBase v7.0 [24], and miRWalk v2.0 [25]. The miRNA-lncRNA interactions are from NPInter v3.0 [26] and LncBase v2.0 [27]. The experimentally validated PPIs are obtained from an integrative interaction database called ConsensusPathDB v32 [28], and the experimentally verified lncRNA-target interactions are from NPInter v3.0 [26], LncRNA2Target v1.2 [29] and LncRNADisease v2015 [30].

A list of breast cancer genes are collected by integrating five databases: COSMIC v77 [31], GAD [32], OMIM [33], BCGD [34] and G2SBC [35]. The list of breast cancer lncRNAs are from LncRNADisease v2015 [30] and Lnc2Cancer v2016 [36]. The list of 40 unique Gene Ontology (GO) terms associated with 10 cancer hallmarks is obtained from Plaisier et al. [37].

### Pipeline of miRSCoPPI

**Fig. 1:**
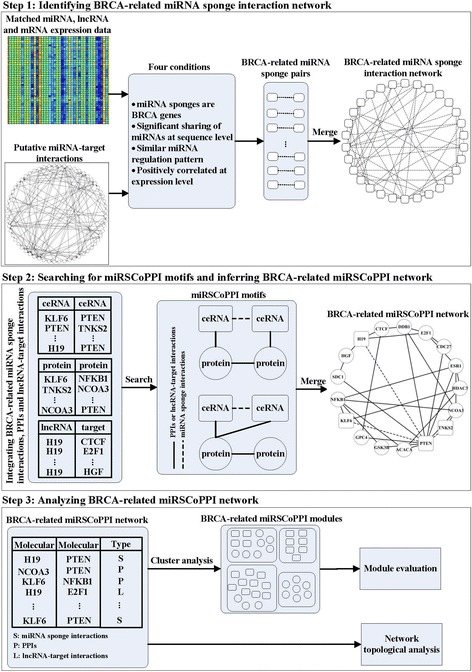
The pipeline of miRSCoPPI. In Step 1, we identify BRCA-related miRNA sponge interaction network by combining matched miRNA, lncRNA and mRNA expression data, and experimentally validated miRNA-target interactions. In Step 2, BRCA-related miRNA sponge interactions, PPIs, and lncRNA-target interactions are firstly combined into a network, and we search for miRSCoPPI motifs from the combined network. Then, the identified miRSCoPPI motifs are merged to obtain the BRCA-related miRSCoPPI network. In Step 3, we analyze the BRCA-related miRSCoPPI network using network topological analysis, cluster analysis and module evaluation

In this section, we propose miRSCoPPI for inferring miRNA sponge co-regulation of PPIs in human breast cancer. As shown in Fig. 1, the method contains the following three steps:

1. Identifying BRCA-related miRNA sponge interaction network. We collect matched expression data of BRCA and putative miRNA-target interactions. By using the expression data of miRNAs, lncRNAs and mRNAs, Pearson correlation of each pair of miRNA-lncRNA and miRNA-mRNA is calculated. Then, we remove the pairs not supported by putative miRNA-target interactions. From these remaining miRNA-lncRNA and miRNA-mRNA pairs, we identify miRNA sponge pairs, i.e., the lncRNA-lncRNA, lncRNA-mRNA and mRNA-mRNA pairs that satisfy the four conditions introduced in the next section. Finally, we merge the identified miRNA sponge pairs to form a BRCA-related miRNA sponge interaction network.
2. Searching for miRSCoPPI motifs and inferring BRCA-related miRSCoPPI network. In this step, we firstly integrate BRCA-related miRNA sponge interactions (obtained from Step 1), PPIs and lncRNA-target interactions into a network. Then we search for two types of miRSCoPPI motifs from the integrated network. Finally, we merge the extracted miRSCoPPI motifs to obtain the BRCA-related miRSCoPPI network.
3. Analyzing miRSCoPPI network. In this step, we conduct network topological analysis and cluster analysis to study the BRCA-related miRSCoPPI network. For each BRCA-related miRSCoPPI module, we also make module evaluation by using functional enrichment analysis and classification analysis.

In the following, we will describe these steps in detail.

### Identifying BRCA-related miRNA sponge interaction network

Given expression data of miRNAs, lncRNAs and mRNAs in BRCA samples, and experimentally validated miRNA-target interactions, with our method, two candidate sponges, ceRNA_*i*_ and ceRNA_*j*_ are accepted as a BRCA-related miRNA sponge pair if all the following four conditions are met:

1. ceRNA_*i*_ and ceRNA_*j*_ are BRCA genes.
  Since the data source used in this work is BRCA dataset, we focus on identifying BRCA-related miRNA sponge pairs where ceRNA_*i*_ and ceRNA_*j*_ must be in the list of breast cancer genes.
2. ceRNA_*i*_ and ceRNA_*j*_ show a significant sharing of miRNAs at sequence level.
  Since the computational complexity of testing all possible combination of RNA-RNA pairs is very high, we only reserve those RNA-RNA pairs with a significant sharing of miRNAs. In this work, we require that ceRNA_*i*_ and ceRNA_*j*_ share at least three miRNAs, and pass the test of significance of the sharing, i.e., the *p*-value obtained from the following hyper-geometric distribution test is less than 0.01.
  1 $$ p=1- F\left( x\Big| M, N, K\right)=1-{\displaystyle \sum_{i=0}^{x-1}\frac{\left(\begin{array}{l} N\\ {} i\end{array}\right)\left(\begin{array}{l} M- N\\ {} K- i\end{array}\right)}{\left(\begin{array}{l} M\\ {} K\end{array}\right)}} $$
  In the formula, *M* is the number of all miRNAs in the dataset, *N* and *K* represent the total numbers of miRNAs regulating ceRNA_*i*_ and ceRNA_*j*_ respectively, and *x* is the number of common miRNAs shared by ceRNA_*i*_ and ceRNA_*j*_.
3. ceRNA_*i*_ and ceRNA_*j*_ have similar miRNA regulation pattern.
  In addition to a significant sharing of miRNAs, ceRNA_*i*_ and ceRNA_*j*_ also should show similar miRNA regulation pattern [38]. Here, we say that ceRNA_*i*_ and ceRNA_*j*_ have similar regulation pattern if the expression levels of them are similarly regulated by their shared miRNAs. If ceRNA_*i*_ and ceRNA_*j*_ have similar miRNA regulation pattern, the two RNAs tend to compete with each other. Thus, we evaluate the similarity of miRNA regulation pattern between two ceRNAs by using two scores, cosine score and collaboration score. Let *M* is the number of common miRNAs shared by ceRNA_*i*_ and ceRNA_*j*_. We compute the Pearson correlation coefficients between the expression levels of each of the *M* common miRNAs and the two ceRNAs (ceRNA_*i*_ and ceRNA_*j*_), *C*_*ik*_ and *C*_*jk*_ (*k* = 1,…,*M*), respectively, then we use the correlation coefficients in the calculation of the cosine score (*Cos*) in the following formula.
  2 $$ C o s\left( ceRN{A}_i, ceRN{A}_j\right)=\frac{{\displaystyle \sum_{k=1}^M{C}_{i k}{C}_{j k}}}{\sqrt{{\displaystyle \sum_{k=1}^M{C_{i k}}^2}}\bullet \sqrt{{\displaystyle \sum_{k=1}^M{C_{j k}}^2}}} $$
  To cater for both up and down miRNA regulations when calculating the collaboration score, we use the absolute values of correlation coefficients (*AC*) between the expression levels of the miRNAs and the ceRNAs as the strength of miRNA-ceRNA interactions. Higher values of *AC* indicate greater strength of miRNA-ceRNA interactions. Then for ceRNA_*i*_, ceRNA_*j*_, based on the strength of the miRNA-ceRNA interactions, the collaboration score (*Col*) of the ceRNA_*i*_-ceRNA_*j*_ pair is calculated as follows:
  3 $$ Col\left( ceRN{A}_i, ceRN{A}_j\right)=\frac{{\displaystyle \sum_{k=1}^M A{C}_{i k} A{C}_{j k}}}{\sqrt{{\displaystyle \sum_{k=1}^M A{C}_{i k}}}\bullet \sqrt{{\displaystyle \sum_{k=1}^M A{C}_{j k}}}} $$
  Since the cosine score and collaboration score is a conservative and excessive estimation of the similarity of miRNA regulation pattern respectively, we regard the average score of the cosine score and collaboration score as the similarity score (*Sim*) of each miRNA sponge pair. To reserve more candidate BRCA-related miRNA sponge pairs, the threshold of *Sim* is set to a moderate value of 0.5.
  4 $$ S i m\left( ceRN{A}_i, ceRN{A}_j\right)=\frac{Cos\left( ceRN{A}_i, ceRN{A}_j\right)+ Col\left( ceRN{A}_i, ceRN{A}_j\right)}{2} $$
4. ceRNA_*i*_ and ceRNA_*j*_ are positively correlated at expression level.
  It has been found that miRNA sponge modulators can decrease the number of free miRNAs available to repress other target genes [13], indicating that the expression levels of miRNA sponge pairs are positively correlated with each other. To identify the active BRCA-related miRNA sponge pairs, we calculate the Pearson correlation of each candidate BRCA-related miRNA sponge pairs. All the candidate BRCA-related miRNA sponge pairs with positive correlations and *p*-value < 0.01 are inferred as BRCA-related miRNA sponge interactions.
  To create the BRCA-related miRNA sponge interaction network, we represent each of the BRCA-related miRNA sponge pairs satisfying the above conditions with two nodes linked together with an undirected edge (denoting their competing interaction), then we merge the linked pairs to get the network.

### Searching for miRSCoPPI motifs and inferring BRCA-related miRSCoPPI network

As shown in Fig. 1, we specify two types of miRSCoPPI motifs by considering the co-regulation of PPIs by two miRNA sponges. Here, the co-regulation between miRNA sponges is determined by whether the two miRNA sponges share a common PPI. This means that the two miRNA sponges each interact with a different party of the same PPI; or the two miRNA sponges may share a common protein that is a party of a PPI.

To search for miRSCoPPI motifs, we firstly integrate BRCA-related miRNA sponge interactions, PPIs, and lncRNA-target interactions into a network. We use the NetMatchStar plugin [39] in Cytoscape [40] to search the integrated network for the two types of miRScoPPI motifs. Finally, we merge the identified miRSCoPPI motifs to obtain the BRCA-related miRSCoPPI network.

### Analyzing miRSCoPPI network

To understand the network topological properties of the BRCA-related miRSCoPPI network, we use the *igraph* R package [41] to analyze the topology of the network. In this study, we treat the edges of the BRCA-related miRSCoPPI network as undirected. In the BRCA-related miRSCoPPI network, the degree of a node is defined as the number of edges connecting the node. The characteristic path length of the BRCA-related miRSCoPPI network is the average of the minimum path lengths (the total number of edges of a path from one node to another node) for all possible pairs of network nodes, and it reflects the compactness of a network. The hub genes with higher degrees in biological networks are more likely to be essential, and it is reported that about 10% of the nodes in a network are essential. To understand the underlying biological implications of hub genes, we use the *clusterProfiler* Bioconductor package [42] to conduct functional enrichment analysis. We are only interested in Gene Ontology (GO) [43] biological processes and Kyoto Encyclopedia of Genes and Genomes (KEGG) [44] pathways at significant level (adjusted *p*-value < 0.01, adjusted by Benjamini-Hochberg method).

To systematically analyze the BRCA-related miRSCoPPI network, we focus on the three types of distributions: node degree, miRSCoPPI motifs and common miRNAs. If a network whose node degree follows a power law model, the network is regarded as scale-free, which is one of most important metric of true biological networks [45]. In addition, if the distributions of miRSCoPPI motifs obey a power law model, then this indicates that most of miRSCoPPI motifs are formed by a minority of BRCA-related miRNA sponge interactions. Similarly, the distributions of common miRNAs following a power law model imply that only a few BRCA-related miRNA sponge interactions share a large number of common miRNAs. Since the distributions of miRSCoPPI motifs and common miRNAs are all associated with BRCA-related miRNA sponge interactions, we further evaluate whether miRSCoPPI motifs and common miRNAs are linearly correlated in the BRCA-related miRSCoPPI network.

For cluster analysis, we use the Markov Clustering Algorithm (MCL) [46] implemented in *ProNet* R package [47] to identify modules in the BRCA-related miRSCoPPI network. To evaluate whether these modules involve in pathways related to breast cancer, we also use the *clusterProfiler* Bioconductor package to conduct KEGG pathway enrichment analysis on the modules.

To further determine if the modules can be module signatures for prognostication or not, we use Support Vector Machine (SVM) [48] with default parameters implemented in the *e1071* R package [49] to evaluate classification performance of the feature genes in each module. We utilize two classification performance metrics: classification accuracy (*ACC*) and area under receiver operating characteristic curve (*AUC*), and make 10-fold cross-validation to evaluate the performance of each module. *ACC* is the number of correct predictions made divided by the total number of predictions made, and can indicate the overall accuracy including true positive rate and true negative rate. *AUC* is equal to the probability that SVM will rank a randomly chosen positive sample higher than a randomly chosen negative one. Here, the modules with high values of *ACC* and *AUC* (i.e., more than 0.9) are regarded as module signatures. To rank the overall performance of the modules, we define a new metric called overall prognostic index (*OPI*) that is the average value of *AUC* and *ACC*. A higher value of *OPI* means better overall classification performance.

## Results

### The topological properties of BRCA-related miRSCoPPI network

We firstly follow Step 1 of miRSCoPPI to infer three types of BRCA-related miRNA sponge interactions (lncRNA-lncRNA, lncRNA-mRNA and mRNA-mRNA), and merge them to form the BRCA-related miRNA sponge interaction network. The network contains 37076 miRNA sponge interactions (details in Additional file 1). Next, according to Step 2 of miRSCoPPI, we infer BRCA-related miRSCoPPI network by merging the found miRSCoPPI motifs. As a result, the BRCA-related miRSCoPPI network contains 11292 miRNA sponge interactions, 10448 PPIs and 165 lncRNA-target interactions (details in Additional file 2).

**Fig. 2:**
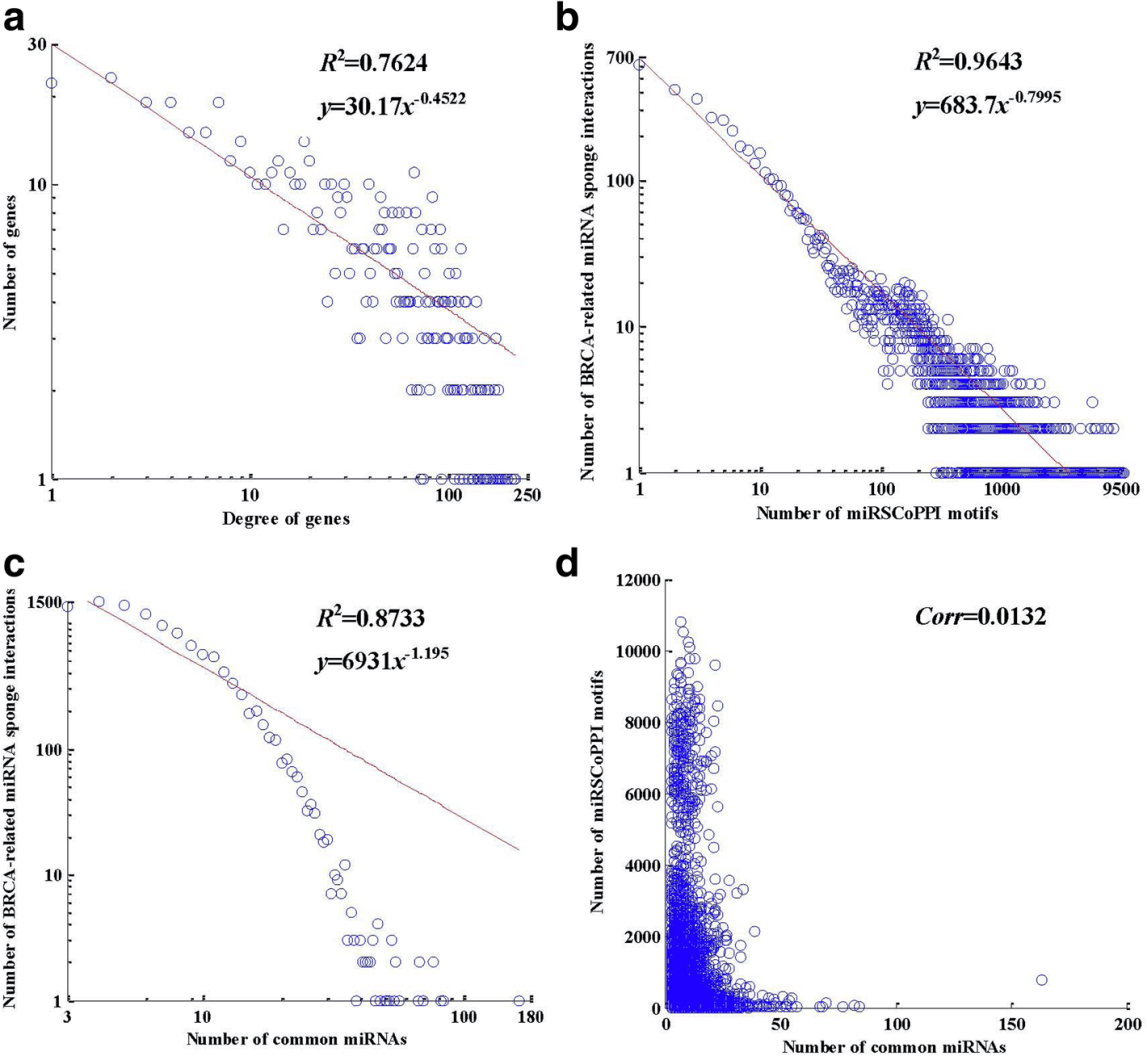
The topological properties of the BRCA-related miRSCoPPI network. **a** Node degree distribution of the BRCA-related miRSCoPPI network. **b** The distribution of miRSCoPPI motifs in the BRCA-related miRSCoPPI network. **c** The distribution of common miRNAs shared by miRNA sponge interactions in the BRCA-related miRSCoPPI network. **d** The correlation between the number of common miRNAs and the number of miRSCoPPI motifs, *Corr* denotes Pearson correlation

The characteristic path length in the network is 2.275, which suggests that the BRCA-related miRSCoPPI network is highly connected and miRNA sponges can promptly co-regulate their downstream protein-protein interactions. The node degree distribution of the BRCA-related miRSCoPPI network fits power law distribution well, with *R* ^2^ = 0.7624 (see Fig. 2a). The result indicates that the BRCA-related miRSCoPPI network is approximately scale free and the topological components such as the hub nodes and modules may have potential biological implications. The distribution of miRSCoPPI motifs formed by BRCA-related miRNA sponge interactions follows power law distribution very well, with *R* ^2^ = 0.9643 (see Fig. 2b). This result implies that most miRSCoPPI motifs are generated by only a minority of BRCA-related miRNA sponge interactions in the BRCA-related miRSCoPPI network. Furthermore, the distribution of common miRNAs shared by each BRCA-related miRNA sponge interactions also obeys power law distribution well, with *R* ^2^ = 0.8733 (see Fig. 2c). The result shows that common miRNAs with large size tend to be shared by a small fraction of BRCA-related miRNA sponge interactions in the BRCA-related miRSCoPPI network. In Fig. 2d, there is no linear correlation between miRSCoPPI motifs and common miRNAs, with *Corr* = 0.0132. In other words, a BRCA-related miRNA sponge interaction with a large number of miRSCoPPI motifs does not necessarily share a large number of common miRNAs, and vice versa.

### Hub genes have potential biological implications in the BRCA-related miRSCoPPI network

Due to the scale-free property, our BRCA-related miRSCoPPI network is thought to be made up of a few highly connected genes. These genes with high node degrees are regarded as hub genes, and may be more essential in the network. In the BRCA-related miRSCoPPI network, we select top 10% of all the genes as hubs (83 genes). To uncover their potential biological implications in breast cancer, we make a functional enrichment analysis of these hub genes using the *clusterProfiler* Bioconductor package.

After functional enrichment analysis, we have discovered that the hub genes are significantly enriched in 337 GO terms and 30 KEGG pathways (details in Additional file 3). According to the list of GO terms mapping to the hallmarks of cancer (details in Additional file 3), 6 out of the 337 GO terms (GO:0045787, GO:0030308, GO:0045786, GO:0090398, GO:0001837, GO:0071456) are involved in 5 cancer hallmarks (Self Sufficiency in Growth Signals, Insensitivity to Antigrowth Signals, Limitless Replicative Potential, Tissue Invasion and Metastasis, Reprogramming Energy Metabolism). Furthermore, several identified KEGG pathways are related to breast cancer, such as Cell cycle (hsa04110) [50], Breast cancer (hsa05224), and Pathways in cancer (hsa05200).

Altogether, the hub genes in the BRCA-related miRSCoPPI network are biologically meaningful, which may imply that our BRCA-related miRSCoPPI network can uncover potential biological implications in breast cancer.

### Modules in the BRCA-related miRSCoPPI network are involved in pathways related to breast cancer

Modules in biological networks may work as functional units underlying complex diseases, including cancer. Thus, we use the MCL clustering method [46] to identify modules in the BRCA-related miRSCoPPI network. Since the size of a miRSCoPPI motif is 4, the minimum module size is set to 4. As a result, we discover 17 BRCA-related miRSCoPPI modules (details in Additional file 4).

**Fig. 3:**
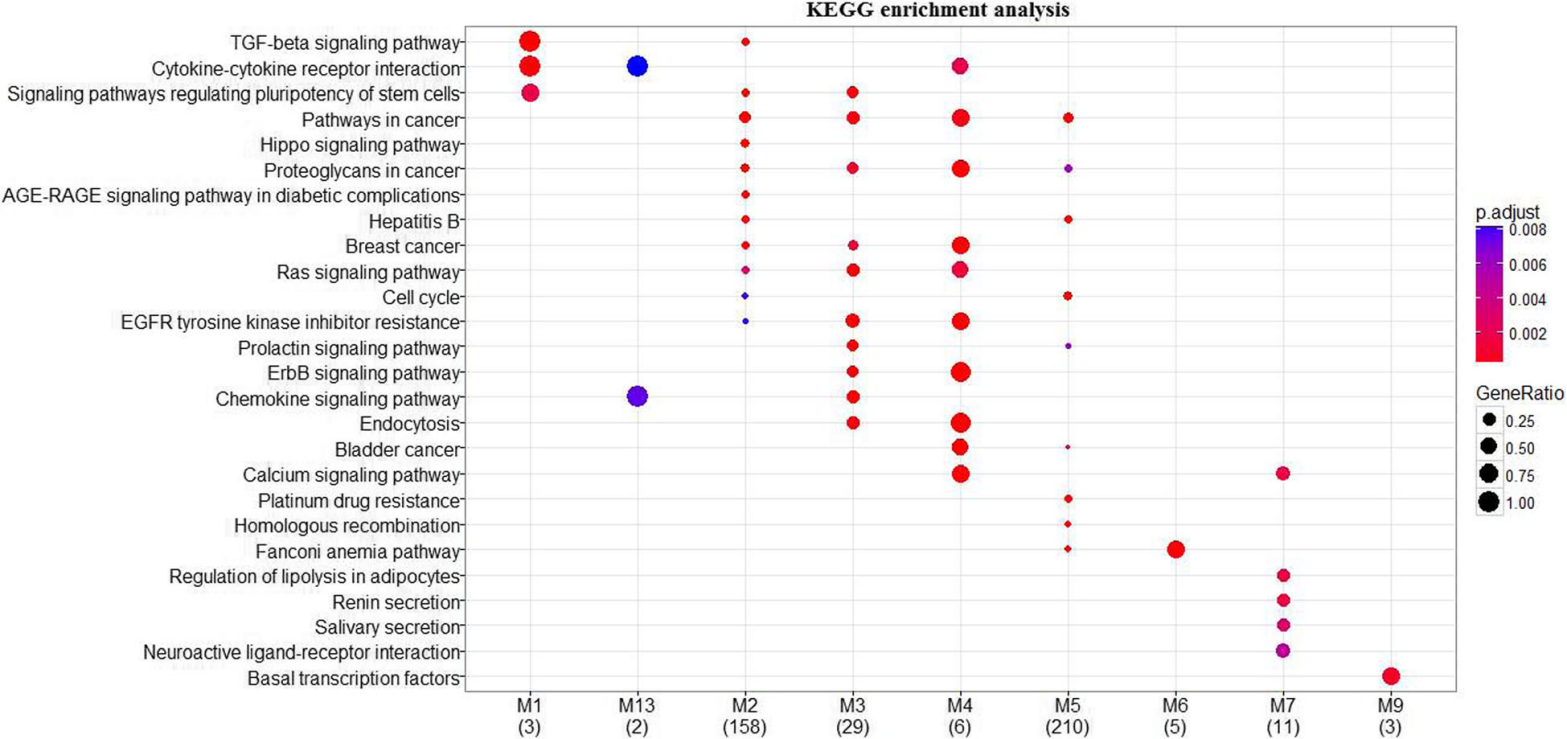
KEGG enrichment results of the BRCA-related miRSCoPPI modules. The number of BRCA-related miRSCoPPI modules having enriched KEGG pathways is 9. The number of horizontal axis is the number of enriched genes. The bubble size indicates the ratio of genes in each term, and different colours correspond to different adjusted *p*-values. The *p*-values are adjusted by Benjamini-Hochberg method

To further understand whether the discovered modules involve in pathways related to breast cancer, we perform pathway enrichment analysis using the *clusterProfiler* Bioconductor package. As shown in Fig. 3, 9 out of 17 modules are significantly enriched in 26 KEGG pathways. Several enriched KEGG pathways are associated with breast cancer, such as Cell cycle [50], TGF-beta signaling pathway [51], Pathways in cancer, Hippo signaling pathway [52], Ras signalling pathway [53], ErbB signaling pathway [54], Chemokine signalling pathway [55], and Calcium signalling pathway [56]. Specifically, three modules (M2, M3, and M4) are significantly involved in the KEGG pathway: Breast cancer, suggesting that the three BRCA-related miRSCoPPI modules are closely associated with breast cancer.

In summary, the pathway enrichment analysis results of BRCA-related miRSCoPPI modules demonstrate that our network is compose of several functional modules related to breast cancer.

### Discriminative modules can act as module signatures in the BRCA-related miRSCoPPI network

As illustrated above, the modules in the BRCA-related miRSCoPPI network are related to breast cancer. Thus, the discriminative modules may act as module signatures. We use SVM classifier to select the discriminative modules that have good performance in classifying human breast tumor and normal samples. As shown in Table 1, 10 out of the 17 discovered BRCA-related modules (58.82%) are regarded as module signatures. The classification performance of each module is ranked in descending order of *OPI*. This result indicates that the 10 BRCA-related miRSCoPPI modules can act as module signatures for prognostication of human breast cancer.

**Table 1:** Module signatures for prognostication

Rank	Module ID	Module size	*AUC*	*ACC*	*OPI*
1	2	291	0.9993	0.9861	0.9927
2	5	335	0.9989	0.9823	0.9906
3	4	7	0.9988	0.9768	0.9878
4	3	35	0.9931	0.9670	0.9800
5	7	12	0.9951	0.9598	0.9775
6	8	4	0.9869	0.9514	0.9691
7	16	8	0.9850	0.9512	0.9681
8	14	7	0.9880	0.9366	0.9623
9	1	5	0.9722	0.9306	0.9514
10	10	7	0.9756	0.9140	0.9448

*AUC*, *ACC* and *OPI* denote classification accuracy, area under receiver operating characteristic curve and overall prognostic index, respectively

**Fig. 4:**
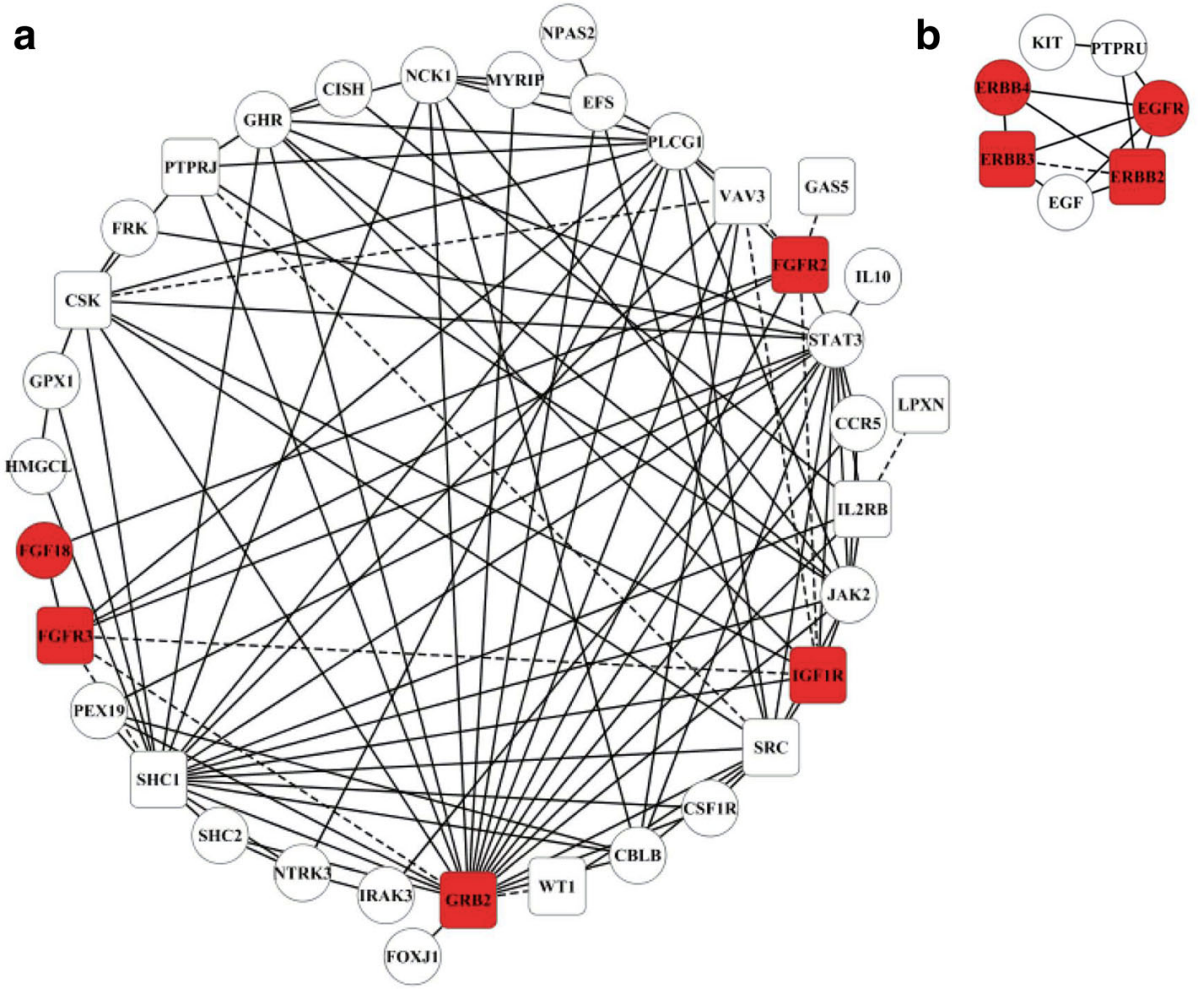
Network presentation of Module 3 (**a**) and Module 4 (**b**). Rectangle and circular nodes indicate miRNA sponges and proteins, respectively. Red rectangle nodes denote known miRNA sponges or proteins from the same family or class. Dashed lines denote miRNA sponge interactions, and solid lines are PPIs

We further analyze Module 2, Module 3 and Module 4 in detail because these modules with good classification performance are also involved in the KEGG pathway: Breast cancer. Figure 4 shows the networks of Module 3 and Module 4. Due to a large number of nodes and edges, the network of Module 2 is not shown and can be seen in Additional file 5. We have discovered that several known miRNA sponges or proteins from the same family or class prefer to cluster in the same module, and interact with each other. In Module 2, there are three families including KLF family (KLF4, KLF5, KLF6, KLF10 and KLF11), SMAD family (SMAD3, SMAD4, SMAD5 and SMAD7), and SOX family (SOX8, SOX9, SOX10 and SOX13). In Module 3 (red nodes in Fig. 4a), FGFR2, FGFR3, FGF18, GRB2 and IGF1R are all growth factors, and EGFR (also called ERBB1), ERBB2, ERBB3 and ERBB4 in Module 4 (red nodes in Fig. 4b) are all from human ERBB family.

### A few BRCA-related miRNA sponge interactions are experimentally validated

**Fig. 5:**
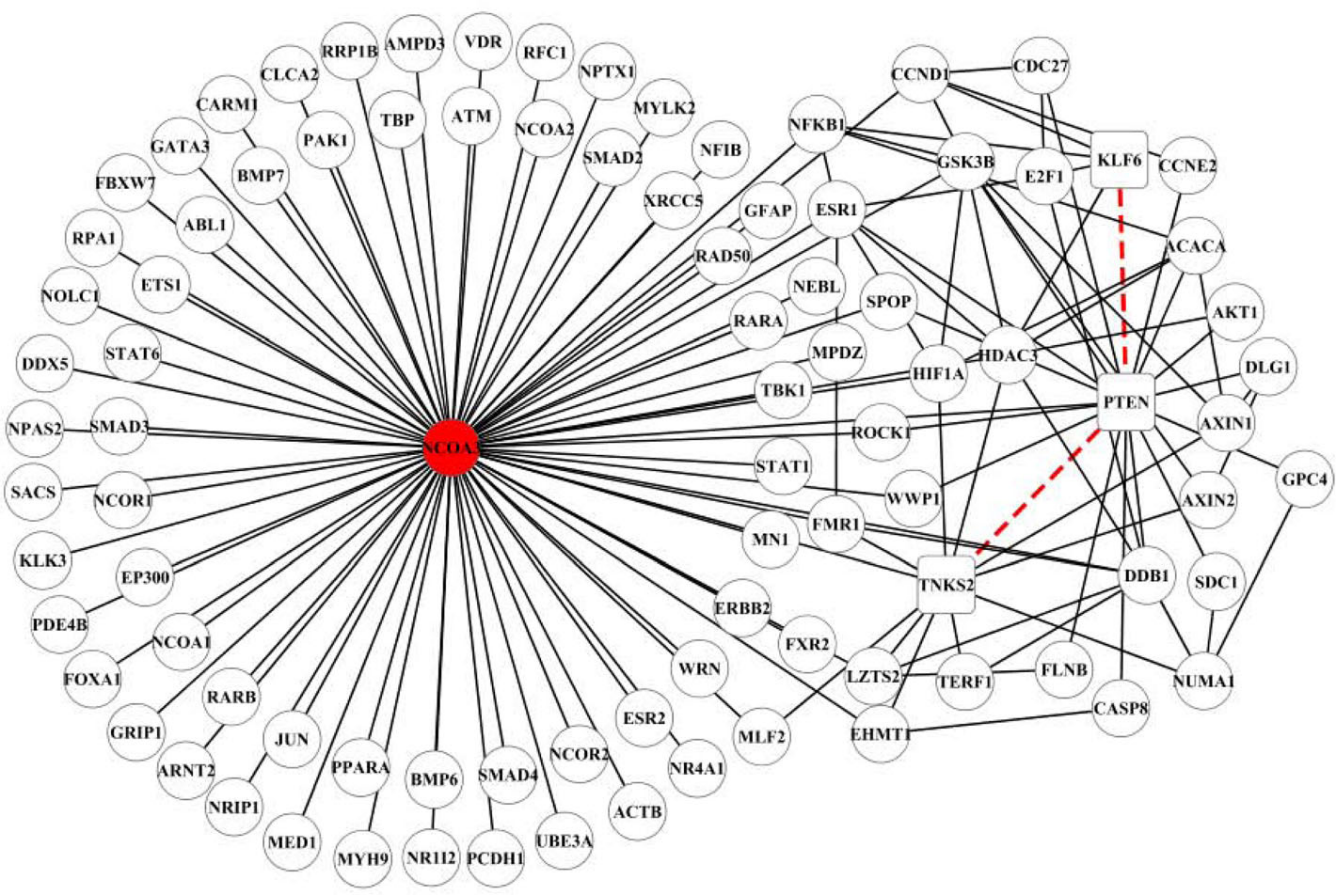
Network visualization of miRSCoPPI sub-network formed by experimentally validated miRNA sponge interactions. Rectangle and circular nodes denote miRNA sponges and proteins, respectively. *Red* circular node denotes the top hub gene in the network. *Red* dashed lines represent experimentally validated miRNA sponge interactions

For the ground truth of validation, we combine the experimentally validated miRNA sponge interactions from [8] with miRSponge [57]. In total, we have obtained 99 experimentally validated miRNA sponge interactions (details in Additional file 6). Only 4 BRCA-related miRNA sponge interactions (H19: IGF2, PTEN:TNKS2, PTEN:RB1, KLF6:PTEN, and “:” stands for competing with each other) are experimentally validated. It is noted that 2 experimentally validated BRCA-related miRNA sponge interactions (PTEN:TNKS2, KLF6:PTEN) can form miRSCoPPI motifs with PPIs. As shown in Fig. 5, the BRCA-related miRSCoPPI sub-network consists of several miRSCoPPI motifs formed by the 2 experimentally validated BRCA-related miRNA sponge interactions. The top hub gene NCOA3 plays an essential role in the network, and the dysregulation of it can disturb the balance of the network and may further cause breast cancer. This result is consistent with the previous finding [58] that NCOA3 disorder is closely associated with breast cancer risk.

### Robustness of the miRSCoPPI method

To evaluate the robustness of the miRSCoPPI method, we use putative computationally predictive miRNA-target interactions to validate the results obtained using experimentally validated miRNA-target interactions. The predictive miRNA-mRNA interactions are obtained from TargetScan v7.1 [59]. The predictive miRNA-lncRNA interactions are from starBase v2.0 [60]. The distributions of node degrees, miRSCoPPI motifs, common miRNAs in the BRCA-related miRSCoPPI network approximately follow power law distribution, with *R* ^2^ = 0.7152, 0.9621, 0.6459, respectively (as shown in Figure S1, Additional file 7). In addition, there is also no linear correlation between miRSCoPPI motifs and common miRNAs, with *Corr* = −0.0681.

In the BRCA-related miRSCoPPI network, we also select top 10% of the genes as hubs (82 genes). The functional enrichment analysis results show that the hub genes are significantly associated with 338 GO terms and 15 KEGG pathways (details in Additional file 3). It is noted that 5 out of 338 GO terms (GO:0045787, GO:0045786, GO:0090398, GO:0001837, GO:0071456) are also related to 5 cancer hallmarks (Self Sufficiency in Growth Signals, Insensitivity to Antigrowth Signals, Limitless Replicative Potential, Tissue Invasion and Metastasis, Reprogramming Energy Metabolism). In addition, several KEGG pathways, such as Cell cycle (hsa04110) [50], Breast cancer (hsa05224), and Pathways in cancer (hsa05200) are related to breast cancer. The results also indicate that the hub genes have potential biological implications in breast cancer.

By using the MCL clustering method, we have discovered 30 BRCA-related miRSCoPPI modules (details in Additional file 4). Fourteen out of 30 modules are significantly associated with 46 KEGG pathways and several pathways are also related to breast cancer (see Figure S2, Additional file 7). Moreover, 22 out of 30 modules (73.33%) have good classification performance and can act as module signatures for prognostication of human breast cancer (see Table S1, Additional file 7).

For the experimental validation of BRCA-related miRNA sponge interactions, only 2 BRCA-related miRNA sponge interactions (PTEN:TNKS2, KLF6:PTEN) are experimentally verified. The 2 BRCA-related miRNA sponge interactions can also form BRCA-related miRSCoPPI sub-network, and the dysregulation of the top hub gene NCOA3 may cause breast cancer [58] (see Figure S3, Additional file 7).

Altogether, the above results are consistent with those obtained using experimentally validated miRNA-target interactions, indicating that miRSCoPPI is robust in inferring miRNA sponge co-regulation of PPIs in human breast cancer.

## Discussion

In this study, we have proposed a multi-step computational method (miRSCoPPI) to infer miRNA sponge co-regulation of PPIs in breast cancer data. Importantly, the BRCA-related miRSCoPPI network follows a power-law distribution, suggesting that most PPIs are co-regulated by a few miRNA sponges. Functional enrichment analysis reveals that hub genes in the BRCA-related miRSCoPPI network have potential biological implications in breast cancer. Several enriched modules are significantly involved in several pathways related to breast cancer. In addition, 58.82% (10 out of 17) of modules may act as module signatures for prognostication due to their good performance in classifying human breast tumor and normal samples. Finally, the proposed method shows robustness in inferring BRCA-related miRNA sponge co-regulation of PPIs.

Crosstalk between miRNA sponges is a novel layer of gene regulation, and it plays vital roles in the physiology and development of human cancers. Therefore, how to infer miRNA sponge interactions is a fundamental question. The basic experimental evidence of miRNA sponge interactions is that miRNA sponge pairs are positively correlated at expression level. In this study, in addition to considering the basic experimental evidence, we also incorporate the similarity of miRNA regulation pattern at expression level into our method to identify miRNA sponge interactions. Although our method focuses on inferring BRCA-related miRNA sponge interactions, the method can also be used to identify generalized miRNA sponge interactions (include tumor and normal-related miRNA sponge interactions) when ignoring the first condition of BRCA-related miRNA sponge pairs.

PPIs are an important part of the entire interactomics system within living cells, and are regarded as functional units of most biological processes including the development and metastasis of human cancers. Thus, investigating the impact of miRNA sponges on PPIs could facilitate the understanding of biological mechanisms of human cancers. The results suggest that miRSCoPPI can be a promising method for inferring miRNA sponge co-regulation of PPIs in human breast cancer.

There are also some limitations in our work. Firstly, miRSCoPPI is a parametric method, i.e., the number of identified miRNA sponge interactions is dependent on the cutoff values of the statistical tests. In this paper, we use the cutoff values that are commonly used in literature, e.g., *p*-value < 0.01. However, changing the cutoff values will result in a change in the set of identified miRNA sponge interactions. Furthermore, due to the lack of available data and the incompleteness of annotations, we do not consider pseudogenes and circRNAs as miRNA sponges in this work. However, the proposed method can be extended in the future when more data is available. Thirdly, due to inadequate knowledge about the co-regulation roles of miRNA sponges on the PPIs, the crosstalks between miRNA sponges and PPIs have not been experimentally validated directly.

## Conclusion

In summary, our study provides a simple pipeline to infer miRNA co-regulation of PPIs that can be applied in different biological areas, such as the study of human cancers and the study of biological processes. We hope that the proposed method is applicable to the study of miRNA sponge co-regulation of PPIs related to other human cancers. Although some limitations exist with the current methods and datasets, the presented results would shed light on the miRNA sponge co-regulation roles in complex human diseases.

## Additional files

Additional file 1: BRCA-related miRNA sponge interaction network. The network includes 37076 miRNA sponge interactions. (XLSX 484 kb)

Additional file 2: BRCA-related miRSCoPPI network. The network contains three types of interactions including 11292 miRNA sponge interactions, 10448 PPIs and 165 lncRNA-target interactions. (XLSX 361 kb)

Additional file 3: Functional enrichment results of hub genes in the BRCA-related miRSCoPPI network. For the experimentally validated miRNA-target interactions, the numbers of enriched GO biological processes and KEGG pathways are 337 and 30, respectively. For the putative computationally predictive miRNA-target interactions, the numbers of enriched GO biological processes and KEGG pathways are 338 and 15, respectively. The *p*-values are adjusted by Benjamini-Hochberg method. (XLSX 86 kb)

Additional file 4: BRCA-related miRSCoPPI modules. For the experimentally validated miRNA-target interactions, 17 BRCA-related miRSCoPPI modules are discovered. Thirty BRCA-related miRSCoPPI modules are discovered for the putative computationally predictive miRNA-target interactions. (XLSX 24 kb)

Additional file 5: BRCA-related miRSCoPPI sub-network in Module 5. The BRCA-related miRSCoPPI sub-network consists of 5075 miRNA sponge interactions, 718 PPIs and 46 lncRNA-target interactions. (XLSX 96 kb)

Additional file 6: Experimentally validated miRNA sponge interactions. The number of experimentally validated miRNA sponge interactions is 99. (XLSX 11 kb)

Additional file 7: Robust evaluation results of the miRSCoPPI method. (PDF 727 kb)

## Abbreviations

ACC: Classification accuracy
AUC: Area under receiver operating characteristic curve
BRCA: Breast invasive carcinoma
ceRNA: Competing endogenous RNA
circRNA: Circular RNA
GO: Gene ontology
KEGG: Kyoto encyclopedia of genes and genomes
KNN: *k*-nearest neighbours
lncRNA: Long non-coding RNA
MCL: Markov clustering algorithm
miRNA: microRNA
miRSCoPPI: miRNA Sponge Co-regulation of PPIs
mRNA: Messenger RNA
OPI: Overall prognostic index
PPI: Protein-protein interaction
SVM: Support vector machine
TCGA: The cancer genome atlas
