# Supplementary material for: Inferring miRNA sponge co-regulation of protein-protein interactions in human breast cancer
Source: BMC Bioinformatics. 2017 May 8;18:243. doi: 10.1186/s12859-017-1672-2 (PMC5423010; doi:10.1186/s12859-017-1672-2)
Supplement: Supplementary file 7 — Robust evaluation results of the miRSCoPPI method. (PDF 727 kb) [file 12859_2017_1672_MOESM7_ESM.pdf]

Inferring miRNA sponge co-regulation of protein-protein interactions in human breast cancer

Junpeng Zhang[1*§], Thuc D Le[2*], Lin Liu[2], Jiuyong Li[2§]

[1]School of Engineering, Dali University, Dali, Yunnan, 671003, P. R. China

[2]School of Information Technology and Mathematical Sciences, University of South Australia, Mawson Lakes, SA 5095, Australia

[§]Corresponding author: zhangjunpeng_411@yahoo.com, jiuyong.li@unisa.edu.au

*These authors contributed equally to this work

In this file, we provide supplementary results of section: **Robustness of the miRSCoPPI method**.

Figures

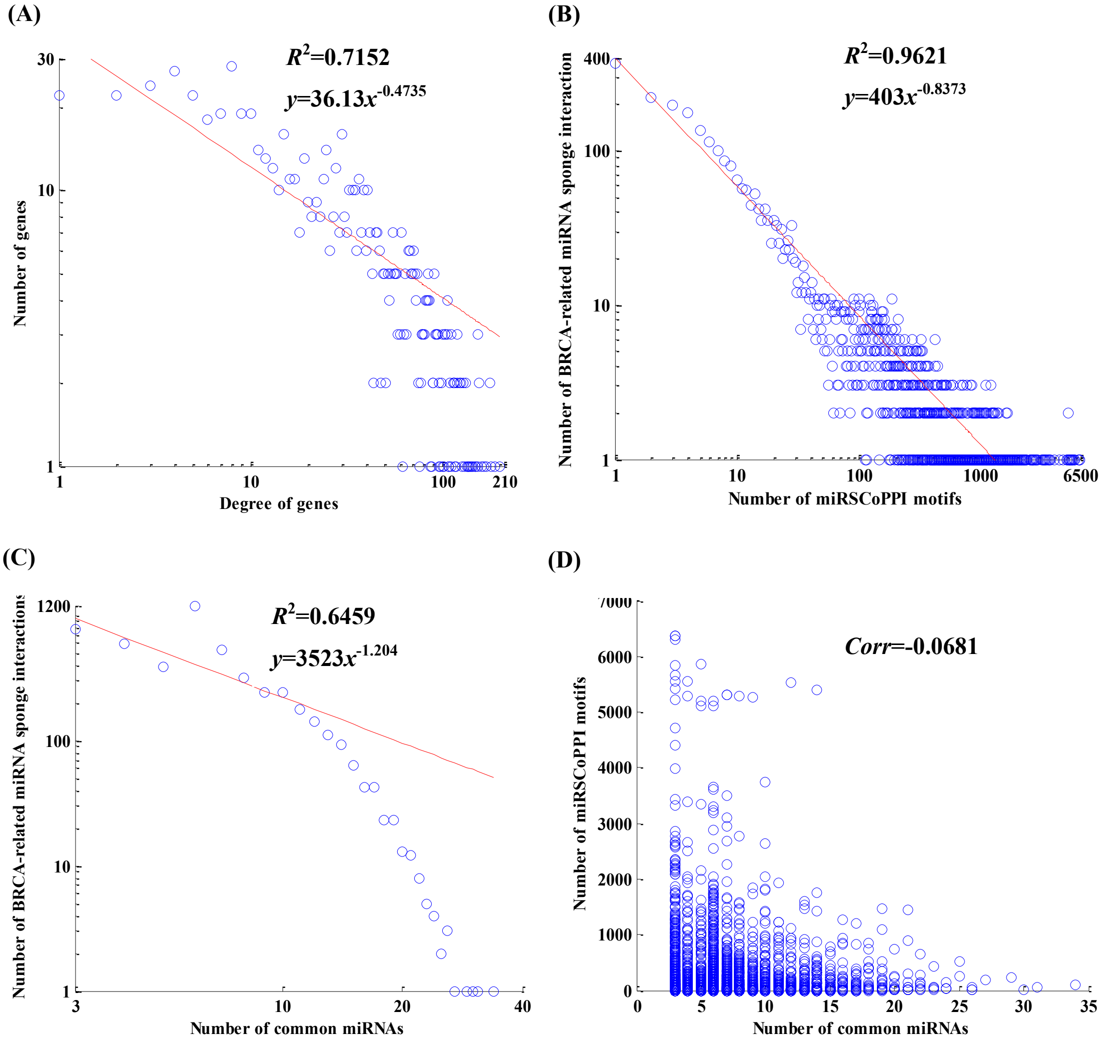

Fig. S1 The topological properties of the BRCA-related miRSCoPPI network. (A) Node degree distribution of the BRCA-related miRSCoPPI network. (B) The distribution of miRSCoPPI motifs in the BRCA-related miRSCoPPI network. (C) The distribution of common miRNAs shared by miRNA sponge interactions in the BRCA-related miRSCoPPI network. (D) The correlation between the number of common miRNAs and the number of miRSCoPPI motifs, *Corr* denotes Pearson correlation. The putative miRNA-target interactions are from TargetScan v7.1 [1] and starBase v2.0 [2].

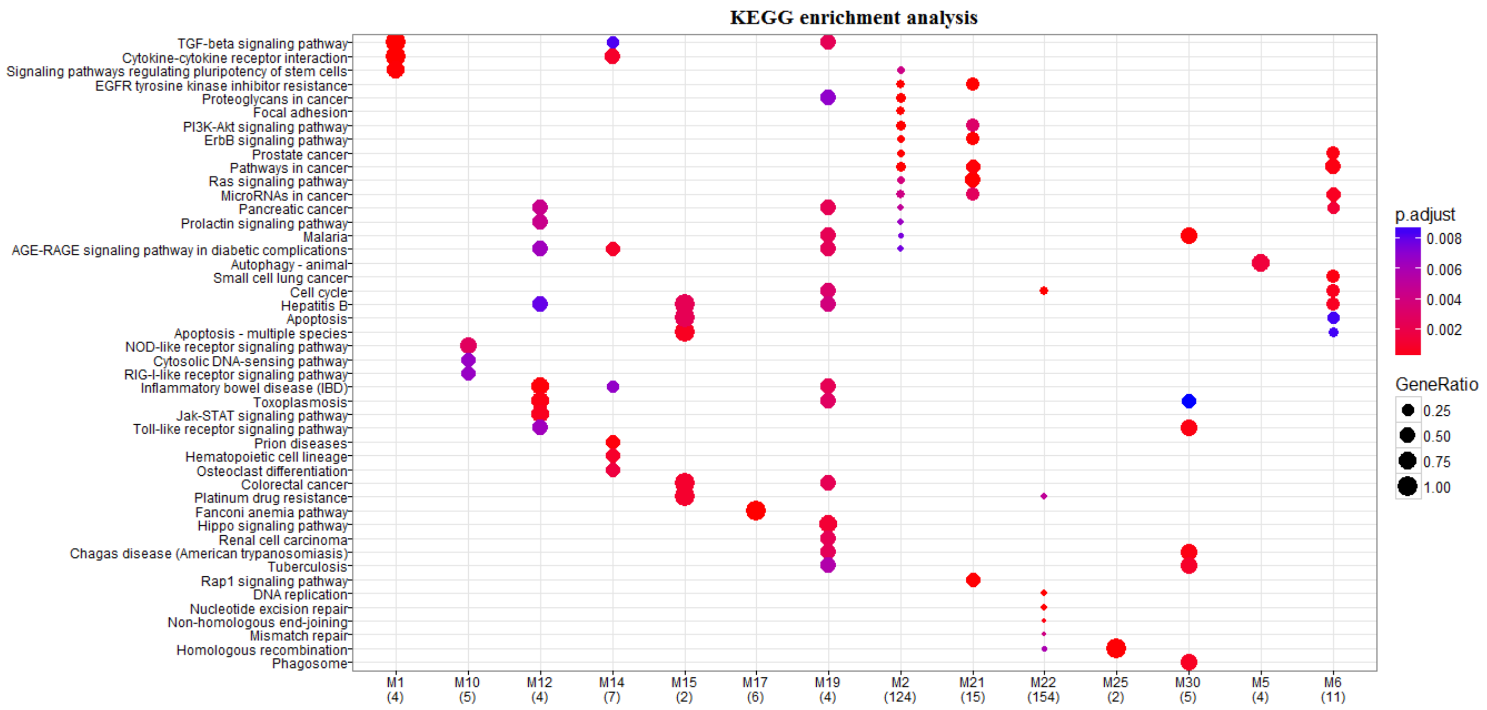

Fig. S2 KEGG enrichment results of the BRCA-related miRSCoPPI modules. The number of BRCA-related miRSCoPPI modules having enriched KEGG pathways is 14. The number of horizontal axis is the number of enriched genes. The bubble size indicates the ratio of genes in each term, and different colours correspond to different adjusted *p*-values. The *p*-values are adjusted by Benjamini-Hochberg method. The putative miRNA-target interactions are from TargetScan v7.1 [1] and starBase v2.0 [2].

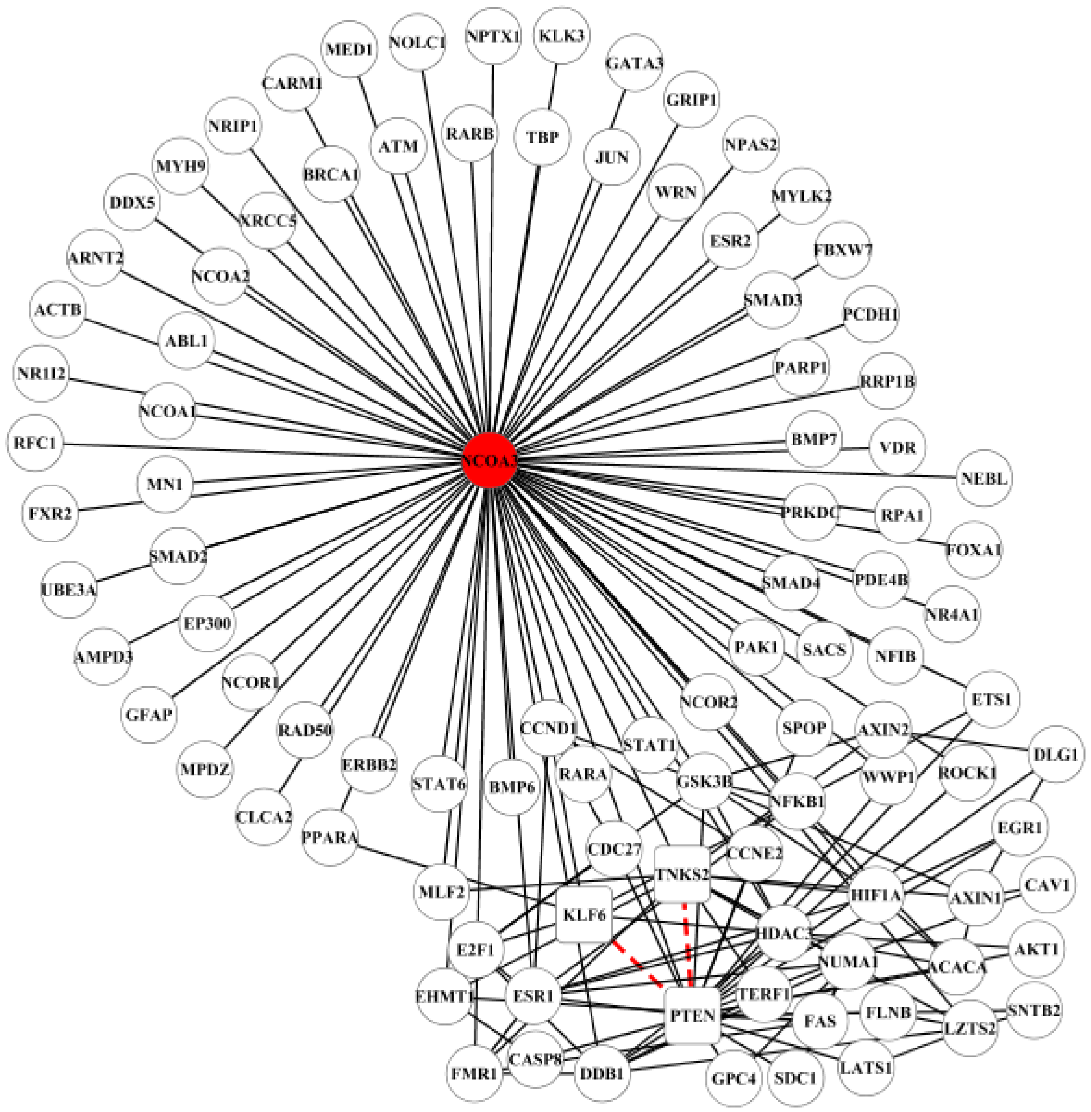

Fig. S3 Network visualization of miRSCoPPI sub-network formed by experimentally validated miRNA sponge interactions. Rectangle and circular nodes denote miRNA sponges and proteins, respectively. Red circular node denotes the top hub gene in the network. Red dashed lines represent experimentally validated miRNA sponge interactions. The putative miRNA-target interactions are from TargetScan v7.1 [1] and starBase v2.0 [2].

Tables

Table 1 Module signatures for prognostication. *AUC*, *ACC* and *OPI* denote classification accuracy, area under receiver operating characteristic curve and overall prognostic index, respectively. The putative miRNA-target interactions are from TargetScan v7.1 [1] and starBase v2.0 [2].

Rank	Module ID	Module size	*AUC*	*ACC*	*OPI*
1	8	27	0.9993	0.9916	0.9955
2	1	6	0.9972	0.9890	0.9931
3	2	197	0.9992	0.9861	0.9926
4	22	270	0.9988	0.9843	0.9915
5	21	23	0.9942	0.9721	0.9831
6	14	12	0.9948	0.9603	0.9776
7	7	13	0.9886	0.9640	0.9763
8	18	25	0.9846	0.9654	0.9750
9	3	5	0.9896	0.9576	0.9736
10	17	6	0.9891	0.9430	0.9661
11	15	5	0.9801	0.9503	0.9652
12	6	14	0.9749	0.9463	0.9606
13	28	4	0.9828	0.9383	0.9606
14	9	6	0.9827	0.9359	0.9593
15	30	5	0.9761	0.9418	0.9590
16	13	8	0.9849	0.9328	0.9588
17	10	7	0.9832	0.9326	0.9579
18	27	8	0.9853	0.9284	0.9569
19	25	4	0.9770	0.9209	0.9490
20	23	4	0.9642	0.9224	0.9433
21	29	4	0.9691	0.9019	0.9355
22	24	7	0.9482	0.9022	0.9252

References

[1] Agarwal V, Bell GW, Nam JW, et al. Predicting effective microRNA target sites in mammalian mRNAs. Elife. 2015; 4:e05005.

[2] Li JH, Liu S, Zhou H, et al. starBase v2.0: decoding miRNA-ceRNA, miRNA-ncRNA and protein-RNA interaction networks from large-scale CLIP-Seq data. Nucleic Acids Res. 2014; 42:D92-7.
